# Use of person-centered goals to direct interdisciplinary care for military service members and Veterans with chronic mTBI and co-occurring psychological conditions

**DOI:** 10.3389/fneur.2022.1015591

**Published:** 2022-11-16

**Authors:** Tracey D. Wallace, Katherine L. McCauley, April T. Hodge, Tim P. Moran, Stephen T. Porter, Maya C. Whaley, Russell K. Gore

**Affiliations:** ^1^SHARE Military Initiative, Shepherd Center, Atlanta, GA, United States; ^2^School of Medicine, Department of Emergency Medicine, Emory University, Atlanta, GA, United States; ^3^School of Medicine, Department of Rehabilitation Medicine, Emory University, Atlanta, GA, United States; ^4^Wallace H. Coulter Department of Biomedical Engineering, Georgia Institute of Technology and Emory University, Atlanta, GA, United States

**Keywords:** concussion, Traumatic Brain Injury, mental health, military, rehabilitation, goals, interdisciplinary, person-centered

## Abstract

**Objective:**

To explore the use of person-centered goals (PCGs) to direct interdisciplinary care to support PCG attainment in military service members and Veterans (SM/Vs) with chronic mild traumatic brain injury (mTBI) and co-occurring psychological conditions.

**Methods:**

A retrospective chart review was completed for 146 United States military SM/Vs reporting chronic symptoms following mTBI and co-occurring psychological conditions who received care in the SHARE Military Initiative intensive outpatient program, a donor-funded program administered by a not-for-profit hospital, between April 1, 2015 and March 31, 2019. PCGs were used to direct care consisting of individual and group-based interventions and therapies delivered by an interdisciplinary, co-located team including behavioral health, case management, neurology or physiatry, nursing, occupational therapy, physical therapy, recreation therapy, speech-language pathology, and transition support. The primary outcome measure was PCG attainment measured *via* goal attainment scaling.

**Results:**

Increased PCG attainment was demonstrated at program discharge and throughout the first year following program discharge. Predictors of goal attainment at discharge included longer participation in treatment, greater reduction in depressive symptoms and greater improvement in adjustment at discharge, male gender, and higher cognitive and physical abilities on admission.

**Conclusions:**

This sample of military SM/Vs with mTBI and co-occurring psychological conditions who received intensive, interdisciplinary, PCG directed care demonstrated increased PCG attainment at program discharge which further increased with transition support over the year post-discharge. Results suggest PGC goal directed care is a feasible, promising methodology of individualizing treatment in this population. This exploratory study lays a foundation for future prospective, controlled, comparative effectiveness research that will further understanding of the effectiveness of intensive, interdisciplinary, PCG directed care.

## Introduction

Traumatic Brain Injury (TBI), the “signature injury” of post-9/11 military service members and Veterans (SM/Vs), can cause significant deficits in physical, behavioral, emotional, social, and cognitive functioning. The Traumatic Brain Injury Center of Excellence reports 453,919 documented TBI's among U.S. service members between 2000 and 2021, the majority of which are classified as mild TBI (mTBI) (1). While most recover from a single mTBI within weeks, as many as 20% of adults have a more prolonged course of recovery, negatively affecting health, function, and participation in important roles (2, 3).

Management of chronic mTBI symptoms in military populations has been a challenge for healthcare systems, in part because of the multifactorial and mutually reinforcing nature of the physical, cognitive, and emotional deficits that require treatment (4–6). Termed the “polytrauma clinical triad,” rates of co-occurrence of TBI, Posttraumatic Stress Disorder (PTSD), and chronic pain are high among SM/Vs (7). Veterans Healthcare Administration records indicate that most Operation Iraqi Freedom/Operation Enduring Freedom/Operation New Dawn Veterans diagnosed with TBI also have a mental health disorder and about half have both PTSD and pain (8). Psychological variables are known predictors of outcomes in the SM/V mTBI population (9, 10).

Specialized, comprehensive care provided through a collaborative multi- or interdisciplinary team approach has been shown to improve outcomes among military populations experiencing chronic effects of mTBI and co-occurring conditions (11–13). Given the heterogeneity of symptom presentations and the potential for individual pre-injury and contextual factors to influence outcomes, rehabilitation outcomes may also be improved by employing person-centered goal (PCG) setting in the context of interdisciplinary care (6, 14). The use of PCGs scaled with goal attainment scaling (GAS) to drive interdisciplinary care has been shown to be efficacious in the management of non-military moderate-severe TBI (15–17); however, this has not previously been examined in military or non-military multidisciplinary management of mTBI (18) with or without psychological comorbidities. PCGs are identified by the person served and address meaningful, motivating aspects of participation and quality of life. GAS is a standardized method of developing a scale to measure progress toward a goal by comparing goal attainment to the person's baseline (19, 20). PCGs scaled by GAS can be used to guide interdisciplinary team collaboration and can support providers in explicitly linking interventions to PCGs when discussing treatments with patients. This model can drive rehabilitation outcomes by empowering patients, motivating helpful behaviors, and connecting treatment engagement to personal goals (21, 22).

Given the intensive resources required to provide PCG directed interdisciplinary care, it is important to evaluate program outcomes and understand factors that contribute to success. This retrospective analysis of military SM/Vs with chronic mTBI and co-occurring psychological conditions who participated in intensive, interdisciplinary, PCG directed care had the following aims: (1) assess changes in PCG attainment following intensive, interdisciplinary, PCG directed care; (2) assess goal attainment in the year following program participation; and (3) identify demographic, injury characteristic, and clinical variables predicting goal attainment. We hypothesized that SM/Vs would report increased goal attainment at program discharge as compared to intake, and that goal attainment would be maintained over time in the year post-discharge. Further, we hypothesized that participant injury characteristics including number of and mechanism of injury, community participation, and psychological functioning including degree of depression, sleep, and PTSD symptoms may predict degree of goal attainment.

## Methods

Institutional review board approval was obtained from Shepherd Center to complete this retrospective chart review.

### Participants

Electronic medical records of a convenience sample of participants who received care in the SHARE Military Initiative (SHARE) intensive outpatient program (IOP), a donor-funded program administered by a not-for-profit hospital, between April 1, 2015 and March 31, 2019 (*n* = 182) were reviewed. Inclusion criteria: (1) unrestricted medical record for review; (2) diagnosis of mTBI assigned during clinical evaluation based on presence of at least one qualifier (23) with patient-report of symptoms persisting greater than 6 months post injury; (3) United States military SM/V; and (4) received treatment in the SHARE IOP. Exclusion criteria were: (1) history of brain injury other than mTBI (*n* = 12); (2) active psychosis (*n* = 0); and (3) discharge PCG GAS ratings unavailable (*n* = 24).

### Intervention

**Figure 1:**
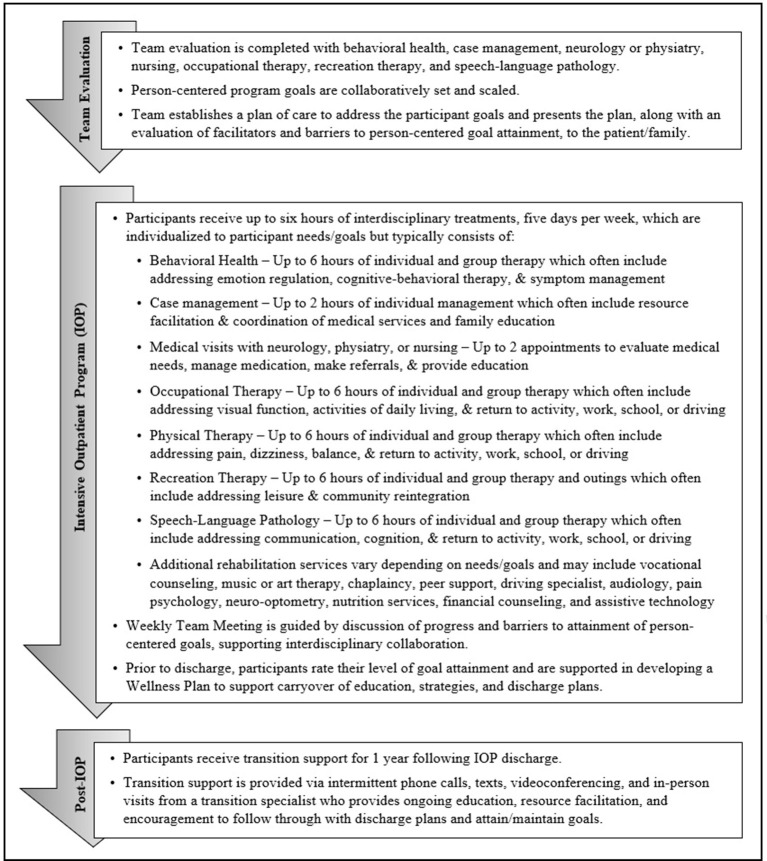
Flow chart of the SHARE Military Initiative intensive outpatient program protocol.

The SHARE IOP provides interdisciplinary outpatient rehabilitation for U.S. SM/Vs experiencing symptoms of brain injury. SHARE IOP participants identify between 1–3 person-centered program goals for which co-located, interdisciplinary team members including behavioral health, case management, neurology or physiatry, nursing, occupational therapy, physical therapy, recreation therapy, and speech-language pathology evaluate facilitators and barriers to develop a comprehensive, PCG directed plan of care (see Figure 1). Program length of stay and the type and amount of therapy provided are individualized given strengths, barriers, symptoms, resources, comorbidities, and other contextual factors unique to each patient (6, 14). Treatment interventions address barriers to goal attainment, and additional services are added (e.g., vocational counseling, art therapy, or chaplaincy) matched to participant PCGs. Program participants receive up to 6 h of individual and group therapy, 5 days per week, delivered by the interdisciplinary team. Following program completion, participants receive support for 1 year from a transition support specialist who provides coaching to adhere to discharge recommendations and maintain goal attainment *via* remote and in-person visits. Program participants collaborate with the care team to apply GAS to their goals on admission (20, 24). Participants then rate their goal attainment at program discharge, and six, nine, and 12 months after discharge.

### Procedures

Two trained researchers conducted chart reviews of medical records to collect demographic, injury, and medical data not electronically abstracted. Number of TBI events were coded as 0, 1, 2, and 3 or more due to ambiguity in records describing greater than three TBIs. Extracted medical record data were recorded into an Excel spreadsheet, and 20% of cases were reviewed by the lead author who measured interrater reliability at 0.96 (Cohen's kappa). Standardized assessment data were electronically extracted for clinical measures administered at admission and discharge. Goal attainment data were electronically extracted for admission, discharge, and timepoints collected post-discharge. Goals were reviewed and categorized by two authors, who first together determined the most common categories based on review of the goals, and then separately assigned each goal to a category. This resulted in discrepancies for 2% of the goals of which 100% were resolved following discussion.

### Measures

Measures are described in Table 1. GAS was used as the primary outcome measure to assess meaningful change in PCGs (19). Participants set program goals and rated goal attainment as outlined above.

**Table 1:** Outcome measures.

**Assessment**	**Objective/description**	**Scoring/assessment type**
**Main outcomes**		
Goal Attainment Scaling (GAS)	Measure meaningful change in person-centered goals for IOP participation	5-point scale collaboratively set by patient and provider according to current status and expected outcomes
**Secondary outcomes**		
Mayo Portland Adaptability Index-4 (MPAI-4)	Assess overall functioning with respect to ability, adjustment, and participation	29-item clinician-rated inventory functioning that yields a total score as well as subscale scores for Adjustment, Ability, Participation
Beck Depression Inventory-II (BDI-II)	Assess depression severity	21-item, self-report inventory
Behavior Rating Inventory of Executive Function –Adult Version (BRIEF-A)	Assess executive function	75-item, self-report inventory that yields a total Global Executive Composite (GEC) score as well as a Behavioral Regulation Index and Metacognitive Index.
Dizziness Handicap Inventory (DHI)	Assess impact of dizziness on function	25-item, self-report inventory
Functional Gait Assessment (FGA)	Assess gait and balance by observing postural stability and the ability to perform multiple motor tasks while walking	10-item, clinician-rated inventory based on observation of gait
Headache Impact Test-−6 (HIT-6)	Assess headache impact on daily function	6-item, self-report questionnaire
Pain Outcome Questionnaire (POQ)	Assess pain	18-item self-report inventory
Pittsburg Sleep Quality Index (PSQI)	Assess sleep	19-item self-report inventory
Post-Traumatic Stress Disorder Checklist for DSM-5 (PCL-5)	Assess severity of post-traumatic stress symptoms	20-item self-report inventory
Post-Traumatic Stress Disorder Checklist—Military Version (PCL-M)	Assess severity of post-traumatic stress symptoms	17-item self-report inventory
Repeatable Battery for the Assessment of Neuropsychological Status Update (RBANS)	Measure change over time in neuropsychological status	Multi item, multi subtest, clinician administered objective measure that yields a Total Score, as well as Immediate Memory, Delayed Memory, Attention, Language, and Visuospatial/ Constructional scores

Clinical measures collected as part of standard care were used in a set of secondary analyses. Clinicians completed Mayo Portland Adaptability Index-4 (MPAI-4) ratings on participants within their first 3 days of program participation, and then again in the last week to evaluate Ability, Adjustment, and Participation as related to body movements, thinking skills, emotions, behavior, and social skills (25). Participants completed clinical measures within their first 2 weeks of program participation and then again in their last week of program participation. Clinical measures included the Beck Depression Inventory-II (BDI-II) (26), Behavior Rating Inventory of Executive Function – Adult Version (BRIEF-A) (27), Dizziness Handicap Inventory (DHI) (28), Functional Gait Assessment (FGA) (29), Headache Impact Test-6 (HIT-6) (30), the Post-Traumatic Stress Disorder Checklist for DSM-5 (PCL-5) (31), Post-Traumatic Stress Disorder Checklist—Military Version (PCL-M) (32), Pittsburg Sleep Quality Index (PSQI) (33), Pain Outcome Questionnaire (POQ) (34), and Repeatable Battery for the Assessment of Neuropsychological Status Update (RBANS) (35) (see Table 1). Some participant records contained PCL-M scores vs. PCL5, which were converted to PCL5 scores for analyses (36).

### Statistical analysis

Categorical and ordinal variables were described using frequencies and percentages. Continuous and scale variables were described using medians and interquartile ranges.

The primary research questions aimed to determine how patient goal attainment, measured using GAS T-scores, changed at program completion and in the year following discharge. A fixed effects linear regression assessment was used to evaluate changes in GAS T-score following discharge by including time since discharge as a predictor using an autoregressive variance/covariance structure to account for multiple measurements per participant. In order to adjust for potential confounding, this analysis was repeated with the following covariates: age, gender, race, ethnicity, education, MPAI-4 subscales measured at admission, change in MPAI-4 subscales from admission to discharge, time since the index TBI, time since the first TBI, time since the most recent TBI, number of known instances of loss of consciousness, BDI-II measured at admission, the change in BDI-II from admission to discharge, the PSQI measured at admission, the change in PSQI from admission to discharge, the PCL measured at admission, the change in PCL from admission to discharge, and the length of stay in the IOP. Both regression coefficients (i.e., the expected change in the outcome per unit change in the predictor in raw units) and standardized regression coefficients (i.e., the expected change in the outcome per unit change in the predictor in standard deviations), as well as 95% confidence intervals, are presented. *P*-values and 95% CIs were computed using bias-corrected and accelerated bootstrapping (10,000 resamples).

In addition to the primary analysis, a number of secondary research questions were considered. First, variation in discharge GAS scores across PCG categories (e.g., fitness-related goals, education-related goals, etc.) were evaluated. Raw GAS values (a 5-point scale) were submitted to a fixed-effects ordinal logistic regression. A fixed-effects ordinal logistic regression was used to account for inclusion of multiple GAS values per participant, as individuals set up to three goals. Goal category was weighted-effects coded so that each odds ratio represents the difference between a given category and the average category. “Other” was chosen as the leave-out category. *P*-values and 95% CIs were computed using bias-corrected and accelerated bootstrapping (10,000 resamples).

We also conducted an exploratory analysis to identify potential predictors of the primary outcome, T-Scores computed from GAS of PCGs measured at discharge (19). In addition to participant demographic and injury characteristics, select clinical variables were included as potential predictors. MPAI-4 scores were included to assess the relationship of functioning commonly impacted by TBI. BDI, PCL-5, and PSQI scores were included to assess the relationship of the severity of symptoms for psychological comorbidities most commonly experienced by SHARE IOP participants, namely depression, PTSD, and sleep difficulties. For each clinical measure, both severity at admission and the degree of change were included in the model. All participants received the same model of care but varying LOS, therefore, LOS was included in the model to determine if the amount of care received influenced goal attainment. Predictors were evaluated using a linear regression and Shapley scores resulting from that analysis. Shapley scores describe the average marginal contribution of each variable to the model output (37). As a result, Shapley scores can be used to evaluate the importance of a given variable with respect to predicting an outcome. Shapley values were scaled so that they sum to 1. Additionally, we present coefficients, standardized coefficients, and 95% confidence intervals.

Finally, an additional analysis evaluated pre-post changes in clinical measures that occurred between admission and discharge. These comparisons were conducted using the Wilcoxon signed-rank test and the standardized mean difference for paired comparisons (i.e., the change from pre- to post expressed in terms of standard deviations). 95% confidence intervals for the standardized mean difference were computed using bias-corrected and accelerated bootstrapping (10,000 resamples).

For the secondary analyses, the false discovery rate was formally controlled (38). Across all variables of interest, a median of 2.7% of data points were missing (mean 11.2%, range 0–58%). The primary outcome, GAS T-Scores, was available for all patients. Ten complete data sets were imputed using fully conditional specification, a multiple imputation method which draws values from each variable's conditional distribution using Markov Chain Monte Carlos (39). All available variables were included in the imputation model. Statistical analyses were conducted using R (v3.6.3) (40).

Because this was a retrospective analysis of existing data, no *a priori* power calculation was conducted. However, the final sample size results in sufficient power (80%) to detect odds ratios >2.6 (assuming maximal variance for a binomial variable), a between-groups standardized mean difference of 0.5 (assuming the minimal asymptotic efficiency of a non-parametric test), a within-groups standardized mean differences of 0.25 (assuming the minimal asymptotic efficiency of a non-parametric test), or a Cohen's f of 0.23.

## Results

The sample included 146 participants who were mostly male (91.8%), with a median age of 37 (IQR 31–44; range 23–60). All participants served in the military post 9–11. Most were either members of the Army (61.6%) or Marines (28.1%) and were separated from service (85.6%). Participants served a median of 10 years (IQR 6–18; range 3–30) and reported a median of two combat deployments (IQR 1–3; range 0–14). The majority had history of multiple TBIs overall (84.4%), multiple blast-TBIs (66.9%), multiple blunt TBIs (51.9%), and at least one injury resulting in loss of consciousness (75.3%). Common co-occurring conditions included sleep disorders (92.5%), PTSD (93.2%), and major depressive disorder (75.3%). Length of program participation ranged from 31 to 148 days (median = 84.5; IQR: 71–98). See Table 2 for details on participant characteristics.

**Table 2:** Participant characteristics.

**Characteristic**	***N* or *M***	**% or IQR**	**Missing in original data (%)**
**Age**, *M*/IQR	37	31–44	0
**Gender**, *N*/%			0
Female	12	8.2	
Male	134	91.8	
**Race**, *N*/%			0
American Indian	4	2.7	
Black	28	19.2	
Multiracial	1	0.6	
White	113	77.4	
**Hispanic**, *N*/%	13	8.9	0
**Marital status**, *N*/%			0
Divorced	16	11.0	
Married	96	65.8	
Separated	10	6.8	
Single	24	16.4	
**Education**, *N*/%			0.4
High school/GED	27	18.5	
Some college/technical school	71	48.6	
Bachelors	32	21.9	
Graduate	16	11.0	
**Military branch**, *N*/%			0
Air Force	10	6.8	
Army	90	61.6	
Navy	11	7.5	
Marines	41	28.1	
National guard	3	2.1	
**Status**, *N*/%			0
Active duty	21	14.4	
Veteran—*Medical Separation*	48	32.9	
Veteran—*Non-Medical Separation*	77	52.7	
**Number of combat deployments**, *M*/IQR	2	1–3	1.9
**Years of service**	10	6–18	0
**Number of blunt TBI**, *M*/IQR	2	1–3	2.7
**Number of blast TBI**, *M*/IQR	3	1–3	0
**Number of known LOC**	1	0–2	10.4
**Years since first TBI**, M/IQR	11	7.5–1	12.7
**Years since index TBI**, *M*/IQR	9	6–12	2.7
**Years since last TBI**, *M*/IQR	7	3.5–11	8.8
**Sleep disorder**	135	92.5	0.8
**PTSD**	136	93.2	0.8
**MDD**	110	75.3	0.8
**GAD**	5	3.4	0.8
**Chronic pain**	25	16.8	0.8
**Somatic symptom disorder**	7	4.7	0.8
**LOS in IOP (days)**, *M*/IQR	84.5	71–98	0

GAD, Generalized Anxiety Disorder; IQR, Interquartile Rang; LOC, Loss of Consciousness; LOS, Length of Stay; M, Median; MDD, Major Depressive Disorder; N, Count; %, Percent.

### Goal attainment

The 146 participants set 281 goals of which 252 were met or exceeded (89.68%) by program discharge. GAS outcomes included goal attainment at much less than expected (0.4%), less than expected (10.4%), expected (32.3%), more than expected (37.3%), and much more than expected (19.65%). The mean T-Score was 57.7 (SD = 9.3) at discharge, indicating better than average goal attainment across participants.

Mean GAS T-Score increased from 57.7 (SD = 9.3) at discharge to 59.5 (SD = 8.2) at 6 months post-discharge, 59.7 (SD = 8.3) at 9 months post-discharge, and 62.8 (SD = 8.5) at 12 months post-discharge. The observed increase in attainment from discharge to 12 months post treatment was significant (β = 0.36, 95% CI: 0.18–0.54, Standardized β = 0.19, *p* < 0.001). This remained significant following adjustment for the covariates listed in Table 3 (β = 0.30, 95% CI: 0.13–0.46, Standardized β = 0.16, *p* < 0.001).

**Table 3:** Predicting discharge goal attainment measured by GAS as a function of demographic and clinical characteristics.

**Predictor**	**β**	**95% CI**	**Std. β**	**Shapley**
**Age**	−0.07	−0.29; 0.16	−0.06	<0.01
**Gender**	-	-	-	0.12
Male	5.82	−0.52; 12.16	0.17	-
Female	Ref	-	-	-
**Race**	-	-	-	0.03
Black	−0.28	−4.43; 3.88	−0.01	-
Other	−2.93	−11.11; 5.26	−0.06	-
White	Ref	-	-	-
**Ethnicity**	-	-	-	<0.01
Hispanic	0.45	−4.82; 5.72	0.01	-
Non-hispanic	Ref	-	-	-
**Education**	1.73	−0.33; 3.79	0.16	0.01
**Admission MPAI-4 ability**	−0.53	−0.98; −0.09	−0.26	0.06
**Admission MPAI-4 adjustment**	0.02	−0.53; 0.57	0.01	0.01
**Admission MPAI-4 participation**	0.56	0.05; 1.07	0.28	0.04
**ΔMPAI-4 ability**	−0.06	−0.45; 0.32	−0.04	0.03
**ΔMPAI-4 adjust**	−0.26	−0.69; 0.16	−0.17	0.11
**ΔMPAI-4 participation**	0.06	−0.43; 0.54	0.03	0.04
**Time since index TBI**	−0.20	−0.77; 0.36	−0.10	0.01
**Time since first TBI**	0.20	−0.12; 0.51	0.16	0.04
**Time since most recent TBI**	0.15	−0.37; 0.68	0.08	0.01
**Number of blunt TBI**	−0.30	−1.69; 1.10	−0.04	<0.01
**Number of blast TBI**	−0.03	−1.43; 1.38	0.00	0.03
**Number of known LOC**	−0.53	−2.38; 1.32	−0.06	0.01
**Admission BDI**	−0.22	−0.45; 0.01	−0.26	0.04
**Admission PSQI**	0.18	−0.33; 0.69	0.07	0.01
**Admission PCL-5**	−0.06	−0.24; 0.12	−0.08	0.02
**ΔBDI-II**	−0.23	−0.41; −0.04	−0.30	0.14
**ΔPSQI**	0.11	−0.37; 0.59	0.04	0.01
**ΔPCL-5**	−0.05	−0.22; 0.12	−0.08	0.04
**LOS in IOP**	0.11	0.03; 0.18	0.24	0.19

### Goal attainment by category

A fixed-effects regression was used to evaluate the relationship between PCGGAS categories and attainment as measured by GAS (see Table 4). Goal categories were weighted-effects coded such that coefficients can be interpreted as the difference between a given goal category and the average goal category. PCGs related to being active in the community were associated with greater GAS improvement (OR = 5.16, 95% CI: 1.94–13.69, *p* =0.001). Improving fitness (OR = 0.37, 95% CI: 0.17–0.83, *p* = 0.02) and success at school (OR = 0.28, 95% CI: 0.14–0.59, *p* = 0.001) were associated with below average GAS achievement.

**Table 4:** Person-centered goal attainment by category type + fixed-effects regression predicting GAS scores as a function of goal categories.

**Goal category**	**Frequency**	**Percent of goals**	**Percent met**	**OR**	**95% CI**	***p***	**Adjusted *p***
Active in community	58	22.3	91.4	5.16	1.94–13.69	0.001^*^	0.006
Family relations	38	14.6	92.1	1.34	0.87–2.05	0.18	1
Behavioral or emotional	36	13.8	83.3	1.17	0.65–2.10	0.60	1
Independence	31	11.9	90.3	0.92	0.38–2.22	0.86	1
Work	24	9.2	95.8	0.83	0.51–1.35	0.45	1
Fitness	23	8.8	73.9	0.37	0.17–0.83	0.02^*^	0.13
Active at home	16	6.2	100	1.10	0.64–1.89	0.74	1
School	15	5.8	80.0	0.28	0.14–0.59	0.001^*^	0.006
Cognitive limitation	12	4.6	91.7	0.96	0.53–1.76	0.91	1
Other	7	2.7	100	Leave-Out Category	-	-	

95% CI, 95% Confidence Interval; OR, Odds Ratio; p, p-value.

* Significant at p < 0.05.

### Predictors of goal attainment

**Figure 2:**
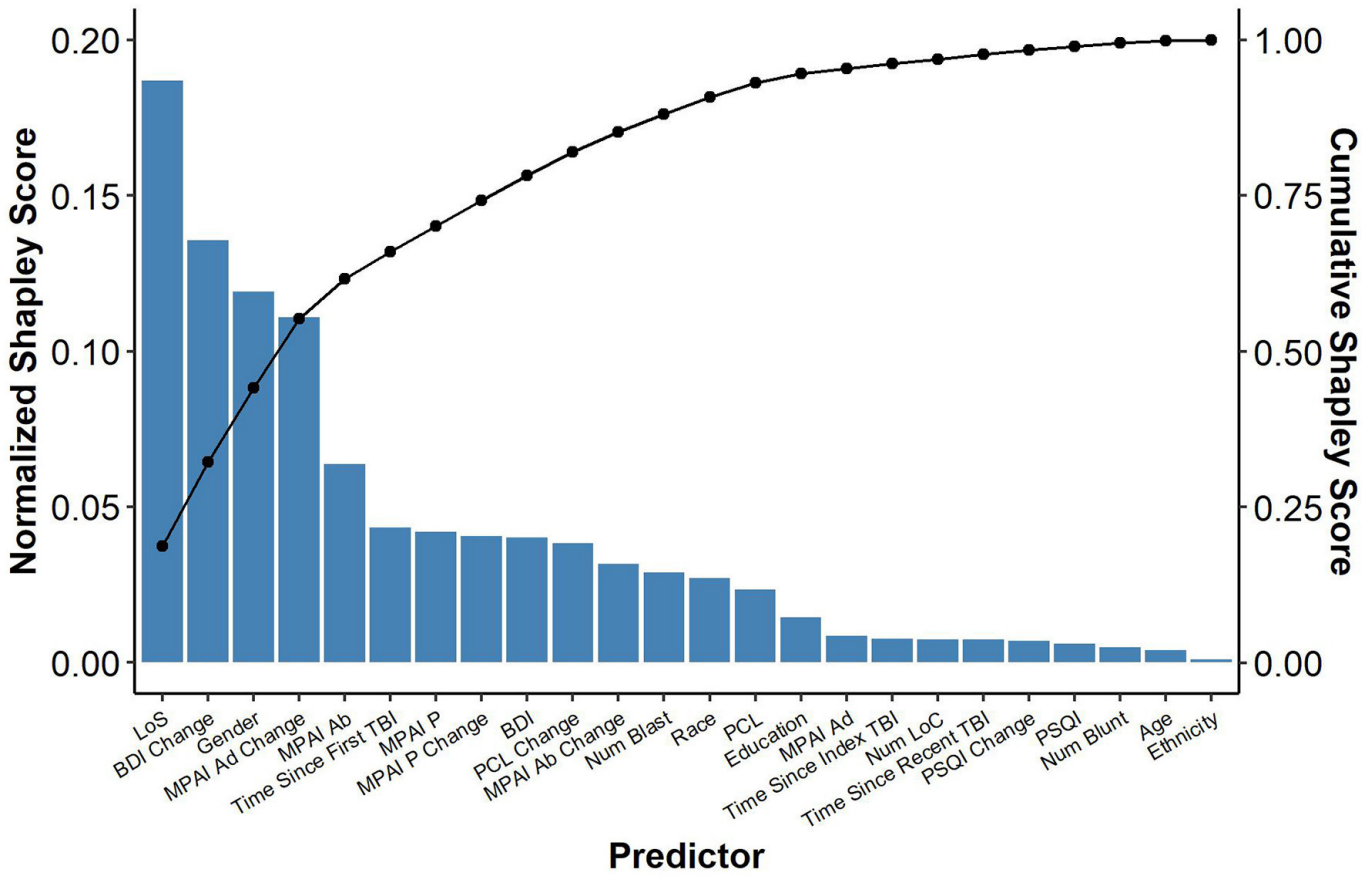
Predictors of goal attainment.

Participant length of stay (longer) in the program was the most important predictor in the model with longer lengths of stay resulting in greater goal attainment (Shapley = 0.19; β = 0.11, 95% CI = 0.03; 0.18, Std. β = 0.24). This was followed by change in BDI-II scores from admission to discharge (Shapley = 0.14; β = −0.23, 95% CI = −0.41; −0.04; Std. β = −0.30), gender (Shapley = 0.12; β = 5.82, 95% CI = −0.52; 12.16, Std. β = 0.17), change in MPAI-4 Adjustment from admission to discharge (Shapley = 0.11; β = −0.26, 95% CI = −0.69; 0.16, Std. β = −0.17), and MPAI-4 Ability measured at admission (Shapley = 0.06; β = −0.53, 95% CI = −0.98; −0.09, Std. β = −0.26) with greater improvement in depression and adjustment, male gender, and higher ability at admission resulting in greater goal attainment. Time since index TBI, MPAI-Participation measured at admission, change in MPAI-Participation from admission to discharge, BDI-II measured at admission, change in PCL from admission to discharge, change in MPAI-Ability form admission to discharge, number of blast injuries, patient race, and PCL measured at admission also made contributions but to a lesser degree. The remaining variables made relatively negligible contributions (see Table 3 and Figure 2).

### Changes in symptoms and community participation

MPAI-4, BDI-II, BRIEF-A, DHI, HIT-6, PCL-5, POQ, and PSQI scores decreased between admission and discharge (all *p* < 0.001), indicating improvement in cognitive and physical abilities, adjustment, community participation, depression, executive function, dizziness, headache, post-traumatic stress symptoms, pain, and sleep. Similarly, RBANS and FGA scores increased between admission and discharge (all *p* < 0.004) demonstrating objective gains in cognitive function and dynamic gait and balance. All comparisons remained significant following the false discovery rate correction (all *p* < 0.01; see Table 5).

**Table 5:** Comparison of clinical measures at admission and discharge.

**Measure**	**Admission** ***M* (IQR)**	**Discharge** ***M* (IQR)**	***p***	**FDR-adjusted *p***	**SMD** **(95% CI)**	**Missing in original (%)**
**MPAI-4**						
Ability	48 (45–51)	37.5 (34–41)	<0.001	<0.001	−1.97 (−2.31; −1.55)	0.4/3.8
Adjustment	54 (52–56)	45 (42–49)	<0.001	<0.001	−1.50 (−1.80; −1.22)	0.4/3.5
Participation	42.5 (40–45.5)	38 (36–41)	<0.001	<0.001	−1.08 (−1.45; −0.74)	0.4/3.1
Total	48 (46–50.5)	37.5 (33–41)	<0.001	<0.001	−1.76 (−2.18; −1.26)	0.4/4.2
BDI-II	32 (22–40)	16 (7.5–22.5)	<0.001	<0.001	−1.15 (−1.34; −0.95)	2.7/23.5
**BRIEF-A**						
BRI	71 (64–78)	59.5 (55.5–63)	<0.001	<0.001	−1.03 (−1.24; −0.80)	14.6/56.9
MI	73.5 (67–80)	60 (56.5–64)	<0.001	<0.001	−1.07 (−1.27; −0.86)	14.2/56.9
GEC	73.5 (67.5–79)	60 (56.5–64)	<0.001	<0.001	−0.98 (−1.23; −0.65)	14.2/56.9
DHI	41 (26–60)	27.5 (14–44.5)	<0.001	<0.001	−0.54 (−0.70; −0.36)	1.2/12.7
FGA	22 (19–26)	28 (25.5–29.5)	<0.001	<0.001	0.82 (0.47; 1.09	10.4/11.2
HIT-6	64 (60–68)	57.5 (52–62.5)	<0.001	<0.001	−0.64 (−0.77; −0.50)	2.3/23.6
PCL-5	52 (46–58)	35 (30–41)	<0.001	<0.001	−1.11 (−1.35; −0.84)	5.4/8.1
POQ	87.5 (69–112.5)	59 (43.5–75.5)	<0.001	<0.001	−0.93 (−1.13; −0.72)	2.3/25.8
PSQI	15 (12.5–17.5)	12 (9.5–14)	<0.001	<0.001	−0.65 (−0.81; −0.48)	9.6/28.1
**RBANS**						
Immediate Memory	81 (72.5–90)	90 (85–97)	<0.001	<0.001	0.61 (0.44; 0.78)	5.4/38.8
Visuospatial/constructional	84 (72–96)	89.5 (82–97)	0.004	0.01	0.22 (0.03; 0.39)	5.4/38.8
Language	87 (78–97)	95 (89–101)	<0.001	<0.001	0.59 (0.42; 0.74)	5.4/38.8
Attention	77.5 (64–91)	87.5 (79.5–96)	<0.001	<0.001	0.54 (0.37; 0.71)	5.4/38.8
Delayed Memory	80.5 (56–94)	88 (81.5–97)	<0.001	<0.001	0.54 (0.37; 0.71)	5.4/38.8
Total	76 (66–87.5)	87.5 (82–93.5)	<0.001	<0.001	0.72 (0.53; 0.91)	5.4/38.8

FDR-Adjusted p, False discovery rate adjusted p-values; GEC, Global Executive Composite; IQR, Interquartile Range; M, Median; MCI, Metacognition Index; p, p-values computed from the Wilcoxon signed-rank test; SMD, standardized mean difference.

## Discussion

This sample of SM/Vs with mTBI and co-occurring psychological conditions demonstrated PCG attainment following participation in intensive, interdisciplinary, PCG directed care. Most participants met or exceeded scaled expectations for PCGs using standardized GAS methodology.

Results demonstrate that goal attainment increased throughout the first year following IOP discharge. The present study design does not allow for evaluation of the impact of post-discharge transition support on outcomes, nor does it consider other healthcare services received in the year following treatment. Nevertheless, maintaining and even improving upon goal attainment after discharge suggests maintenance and translation of gains post treatment into home and community environments. Further, while maintenance of gains following intensive, interdisciplinary TBI rehabilitation has been previously demonstrated (13, 41), an increase in treatment gains has only previously been reported after intensive rehabilitation within 6 months of TBI (42).

The most important predictors of goal attainment at program discharge were longer length of stay in the treatment program greater reduction in depressive symptoms and greater improvement in adjustment at discharge, gender, and higher cognitive and physical abilities on admission.

Longer length of stay was the most important predictor of greater goal attainment. This is consistent with other studies examining moderate and severe TBI outcomes which found that longer treatment course and intensity of services positively correlate with improved function and increased community re-integration (43, 44). Repetitive task-specific training drives plasticity leading to improved outcomes in neurological populations (45), and a longer length of stay may facilitate the repetitions and practice needed to create change and make functional improvements. A longer stay may also permit more opportunities to practice use of compensatory strategies in different functional contexts, increasing potential for mastery.

Greater reduction in depressive symptoms and greater improvement in adjustment were also important predictors of greater goal attainment. Behavioral theories of depression posit a bidirectional relationship between mood and activity levels, such that those who are depressed engage in less rewarding activity, which in turn maintains or worsens depression (46, 47). The interdisciplinary rehabilitation included interventions and experiences known to improve depression and adjustment, such as Cognitive Behavioral Therapy skills (48), behavioral activation (49), community outings and engagement in leisure activities targeting self-efficacy and environmental reward (46), contact with supportive peers (50), and increased physical activity (51). Given the participation-oriented nature of many PCGs, it is possible that improvements in depression supported attainment, but also that simultaneously addressing cognitive, emotional, and physical mTBI symptoms that pose barriers to community participation and behavioral activation led to both goal attainment and improvement in mood and adjustment. Future prospective research could help ascertain the nature of the relations between change in overall psychological well-being and goal attainment.

Gender was identified as an important variable predicting goal attainment with male gender resulting in greater goal attainment. This finding may have limited generalizability given the small number of females in the sample. However, other studies have found poorer outcomes to be associated with female gender in chronic mTBI (52, 53) and more research is needed to understand the specific healthcare needs of females post mTBI. Higher cognitive and physical abilities as measured by the MPAI-4 Ability score at admission was also an important predictor. Given that higher cognitive functioning supports goal directed behavior (54), participants with higher cognitive abilities on admission may have been more engaged by the PCG directed care model, thereby leading to greater goal attainment. Higher cognitive functioning may also be supportive of greater success implementing other rehabilitation strategies and interventions (55). Both higher cognitive and physical abilities may be indicative of lower global impairment, thereby contributing to more favorable outcomes for those participants (56). Further, 52.3% of goals identified by participants targeted activity and participation (e.g., active in the community, fitness, and active at home) with active in the community being the most common category of goals set and the category associated with greatest goal attainment, and it is likely participants with greater abilities experienced fewer barriers to increasing activity and participation. Further study is necessary to determine specific subgroups and injury characteristics that benefit most from PCG and GAS focused management.

An exploratory analysis of clinical measures revealed participants also demonstrated improvements in measures assessing participation, as well as cognitive (e.g., memory, attention, executive function), psychological (e.g., depression, PTSD, sleep), and physical (e.g., pain, headaches, dizziness) impairment. These results cannot be interpreted as resultant of the treatment intervention given the lack of a control group. However, gains were significant in these pre-post comparisons. MPAI-4 results were in the mild to moderate impairment range on admission (40–50) and improved to the mild impairment range at discharge (30–40) (57). In other TBI studies, this degree of change correlated with improved independence, return to work, and goal attainment (16, 58). The observed increased PCG and improvements across symptom and participation measures are notable because all participants were experiencing chronic mTBI symptoms, many of them for years post injury, and the sample includes participants with potentially more severe injuries and/or comorbidities given that one-third were medically separated from service.

### Study limitations

This study is marked by some limitations that should be noted in interpretation of these results. A limitation of the study design is a lack of control condition, and accordingly these findings do not definitively conclude that the intervention used resulted in the improvements noted. Improvements demonstrated in pre-post comparisons may be attributed to factors unrelated to the intervention, such as response to attention given by clinicians or spontaneous recovery. Further, generalizability of findings is limited by use of convenience sampling. Generalizability to other programs is also limited by the setting. While the SHARE IOP services SM/Vs, the program takes place in a civilian setting and may not be generalizable to military settings where SM/Vs more frequently seek care. Likewise, while the results may not be generalizable to non-military individuals with mTBI and co-occurring psychological conditions. Data for these analyses were obtained through retrospective chart review, and much was extracted from narratives in progress notes; therefore, it is probable that this data is incomplete. Injury count data was coded as, “0, 1, 2, 3 or more,” because often at the point of three or more of a type of TBI mechanism (blast, blunt force), it was unclear exactly how many were sustained. Many outcome measures were self-reported, which can introduce bias. Patient reported measures were collected by the participants' clinical providers, which may have introduced a reporting bias in which some participants may have rated their improvement as greater in order to please their providers. In addition, RBANS gains may be resultant of practice effects given the short time period between test administration. While the hypothesis tests included in the regression were conducted a priori, it did involve a large number of comparisons which may result in an elevated rate of false positives. Further, several predictors in the regression model were non-significant. While it is possible that these predictors do not contribute to goal attainment, it is also possible that the sample size resulted in insufficient power to detect these effects. The study sample was comprised predominantly of younger White males, and findings may not generalize to more diverse samples of SM/Vs. Finally, the impact of medications on treatment outcomes was not considered and could account for some of the variance in outcomes.

## Conclusion

This sample of SM/Vs with mTBI and co-occurring psychological conditions demonstrated increased goal attainment following intensive, interdisciplinary, PCG directed care which further increased with transition support over the year post-discharge. Results suggest PGC goal directed care is a feasible, promising methodology of individualizing treatment in this population. The retrospective chart review design used in this study does not allow us to determine which components of the care model influenced outcomes, but this exploratory study lays a foundation for future prospective, controlled, comparative effectiveness research that can further our understanding of the effectiveness of intensive, interdisciplinary, PCG directed care. Participant goal attainment mirrored improvements demonstrated on traditional clinical measures of TBI outcomes, suggesting PCG directed care is a promising methodology of individualizing treatment in this complex patient population and should be further explored in both military and non-military mTBI.

## Data availability statement

The raw data supporting the conclusions of this article will be made available by the authors, without undue reservation.

## Ethics statement

The studies involving human participants were reviewed and approved by the Shepherd Center Research Review Committee (RRC). A waiver of written informed consent was granted by the RRC, and abstracted medical records did not contain those who opted out of outcomes research at the time of registration.

## Author contributions

TW and RG conceptualized the study. TW, AH, SP, and RG deigned the study. TW, AH, SP, and MW collected the data. TW, KM, AH, TM, SP, MW, and RG analyzed and interpreted the data and drafted the manuscript. TW, TM, and MW designed the tables and figure. All authors contributed to the article and approved the submitted version and agree to be accountable for the content of the work.

## Funding

Open access publication fee funding provided by Shepherd Center.

## Conflict of interest

The authors declare that the research was conducted in the absence of any commercial or financial relationships that could be construed as a potential conflict of interest.

## Publisher's note

All claims expressed in this article are solely those of the authors and do not necessarily represent those of their affiliated organizations, or those of the publisher, the editors and the reviewers. Any product that may be evaluated in this article, or claim that may be made by its manufacturer, is not guaranteed or endorsed by the publisher.
